# Asymmetrical Handgrip Strength Is Associated with Lower Cognitive Performance in the Elderly

**DOI:** 10.3390/jcm11102904

**Published:** 2022-05-20

**Authors:** Ju-Young Choi, Sohyae Lee, Jin-Young Min, Kyoung-Bok Min

**Affiliations:** 1 Department of Preventive Medicine, College of Medicine, Seoul National University, Seoul 03080, Korea; cjuyoung@snu.ac.kr (J.-Y.C.); leesohyae@snu.ac.kr (S.L.); 2 Veterans Medical Research Institute, Veterans Health Service Medical Center, Seoul 05368, Korea

**Keywords:** asymmetric, handgrip strength, cognitive impairment, risk factor, older people

## Abstract

(1) Background: Several studies have reported that handgrip strength (HGS) may be a sign of lower cognitive performance. However, studies supporting an association between asymmetrical HGS and cognitive function are lacking. This study aimed to determine the association between asymmetrical HGS and cognitive performance among the elderly. (2) Methods: The study sample included 2729 individuals aged ≥60 years-old who participated in the 2011–2014 National Health and Nutrition Examination Survey. The cognitive tests consisted of the word learning and recall modules from the Consortium to Establish a Registry for Alzheimer’s Disease (CERAD), Animal Fluency Test, and Digit Symbol Substitution Test (DSST). HGS was measured using a handgrip dynamometer, and asymmetrical HGS was used to calculate HGS. (3) Results: Of the 2729 participants, 53.0% were aged 60 to 69 years-old, and 47.0% were aged 70 years and older. All cognitive performance scores were significantly correlated with asymmetrical HGS in both age groups. After adjusting for confounders, there was a significant association between DSST and HGS asymmetry in both age groups. Contrastingly, a significant association was only observed for the relationship between the CERAD test and HGS asymmetry in the ≥70 year-old group. (4) Conclusions: We found that low cognitive function was associated with asymmetrical HGS in elderly participants in the United States. Thus, asymmetrical HGS may be an important predictor of cognitive deficits. However, further research is required to confirm our results and to establish possible mechanisms.

## 1. Introduction

The global population is rapidly aging, and the number of people aged 65 or over is expected to increase from 703 million in 2019 to 1.5 billion in 2050 [1]. With the increasing older population, age-related cognitive impairment has increased and will become a public health problem unless prevention and intervention occurs [2]. Cognitive impairment affects quality of life, personal relationships, and health care needs [3]. Moreover, dementia resulting from cognitive decline is irreversible, and there are no specific treatments or drugs for dementia [4]. Thus, it is important to develop ways to reduce the risk of low cognitive performance. As a result, several recent studies have focused on identifying risk factors for cognitive decline. Risk factors associated with low cognitive function include older age, education [5], smoking status [6], alcohol consumption [7], shortened sleep duration [8], and physical activity [9].

Handgrip strength (HGS) has been widely used to estimate frailty, risk of falls [10,11], and muscle mass in clinical and epidemiological studies among the elderly [12,13]. Although HGS is generally considered a measurement of physical or motor function, studies have demonstrated a relationship between HGS and cognition [14,15,16]. Low cognitive function and muscle weakness predict detrimental health conditions such as performance problems (e.g., activities of daily living, trips somewhere, determining a route, and dialing a phone number) and mortality in older people [17,18,19]. Furthermore, HGS is an indicator of physical function, including muscle mass and status, particularly among the elderly [20], and is related to cognitive impairment in longitudinal studies [21,22]. As a result, some studies have suggested measures for using HGS to examine the risk of dementia or Alzheimer’s disease [23,24]. HGS predicts a decline in cognitive performance, such as spatial ability, short-term memory, verbal memory, and processing speed [15,25].

One interesting question is whether asymmetrical HGS is related to cognitive function. Hand dominance often reflects brain hemisphere dominance [23], and a wide range of asymmetrical HGS may reflect morbidity-related dysfunction in the brain hemisphere [24]. Given that differences between hands are associated with adverse health conditions, such as physical frailty and neurophysiological cognitive problems among the elderly, the degree of asymmetrical HGS may be a sign of worse performance in cognitive domains. Recent studies have shown that subjects with asymmetrical HGS have greater odds of functional limitations and lower cognitive performance than those with symmetrical HGS [26,27]. However, there is a lack of evidence supporting an association between asymmetrical HGS and cognitive function. Therefore, this study aimed to investigate whether asymmetrical HGS is associated with cognitive performance in the elderly population.

## 2. Materials and Methods

### 2.1. Study Population

The National Health and Nutrition Examination Survey (NHANES) is a nationally representative survey of the non-institutionalized civilian population in the United States conducted by the Center for Disease Control and Prevention. Publicly available data on grip strength and cognitive function tests were collected from the 2011–2012 and 2013–2014 waves of the NHANES. The National Center for Health Statistics Institutional Review Board [28].

A total of 3632 participants aged ≥60 years-old (range: 60–80 years) were initially selected. Cognitive function tests were conducted on participants aged ≥60 years-old who understood or read the questionnaires in one of the languages provided. Among these individuals, we excluded 483 subjects who had no data for the Consortium to Establish a Registry for Alzheimer’s Disease (CERAD) test. Another 420 participants with missing data on other variables (i.e., income, alcohol consumption, moderate recreational activities, body mass index (BMI), and disease history information) were excluded. The final sample size was 2729 participants.

### 2.2. Measurement of Cognitive Function

The NHANES cognitive function questionnaire included the CERAD test, animal fluency test (AFT), and digit symbol test (DSST) for all participants. Of these cognitive functioning assessments, the CERAD word learning sub-test evaluated the immediate and delayed learning ability for new verbal information, which is the memory sub-domain. The CERAD test consists of three consecutive learning trials. Participants were asked to read 10 unrelated words when they were presented one at a time, and the order of the 10 words was changed in each of the three learning trials. Immediately following the presentation of the words, participants were instructed to recall as many words as possible. The final score for the CERAD test was the sum of three trials, and the maximum score ranged from 0 to 10 in each trial. The AFT, a component of executive function, assesses categorical verbal fluency. The score for named animals and the total scores for the AFT were recorded by presenting the name of as many animals as possible in one minute. DSST, a performance module from the Wechsler Adult Intelligence Scale, Third Edition, was used to process speed, sustained attention, and working memory. These tests were conducted in paper form with a key at the top, including nine numbers matched with symbols. Participants were asked to copy the corresponding symbols in 133 boxes adjacent to the numbers within 2 min. The final score was the sum of the number of correct matches, ranging from 0 to 133. The higher the score, the better the cognitive function.

### 2.3. Measurement of HGS

HGS was measured by a trained research assistant using a Takei digital grip strength dynamometer (Takei Dynamometer Model T.K.K.5401; Akiha-Ku, Japan). Each dominant and non-dominant hand was tested three times, with 60 s of rest of the same hand while alternating hands. The study used the average HGS values for three trials on each hand. HGS asymmetry was calculated as the HGS ratio [dominant HGS (kg)/non-dominant HGS (kg)] [26].

### 2.4. Other Variables

Questionnaire information included age (60–69 or ≥70 years-old), sex (male or female), race/ethnicity (non-Hispanic white, non-Hispanic black, Hispanic, or other), and annual household income (<USD 20,000 or ≥USD 20,000). Health behavior variables included smoking status (current, former, or never), alcohol consumption (drinker or non-drinker), moderate recreational activities (yes or no), and BMI. BMI was calculated by dividing the individual’s weight (kg) by his or her height squared (m^2^) and categorized into the following four groups: underweight (<18.5 kg/m^2^), normal weight (18.5–22.9 kg/m^2^), overweight (23–24.9 kg/m^2^), and obese (≥25 kg/m^2^). Disease history information included physical diagnoses of diabetes (yes or no) and hypertension (yes or no).

### 2.5. Statistical Analyses

Statistical differences among the study population characteristics based on age group (60 or ≥70 years-old) were analyzed. For each variable, a chi-square test was performed for significance testing in the subject groups. Pearson’s correlation coefficients between cognitive function and the HGS were also calculated. Linear regression analysis evaluated the association between HGS and cognitive function tests in each age group and provided beta coefficients and standard errors (SE). Additionally, regression models were adjusted for age, sex, ethnicity, household income, smoking status, alcohol consumption, moderate recreational activities, BMI, and a history of diabetes and hypertension.

This study aimed to obtain weighted estimates of population parameters based on the NHANES analytic and reporting guidelines. All statistical analyses were performed using the PROC SURVEY procedures in SAS 9.4 (SAS Institute, Cary, NC, USA), and statistical significance was set at α = 0.05.

## 3. Results

### 3.1. Characteristics of Participants

Table 1 summarizes the characteristics of the study population according to the two age groups. Of the 2729 participants, 1446 (53.0%) were aged 60 to 69 years-old, and 1283 (47.0%) were aged 70 years and older. Among ethnicity, cigarette smoking, alcohol consumption, moderate activities, BMI, and hypertension, there were significant differences between the groups. However, these data did not show any significant differences in sex, income, or diabetes.

### 3.2. The Structures of Right and Left HGS and Handgrip Ratio

**Figure 1:**
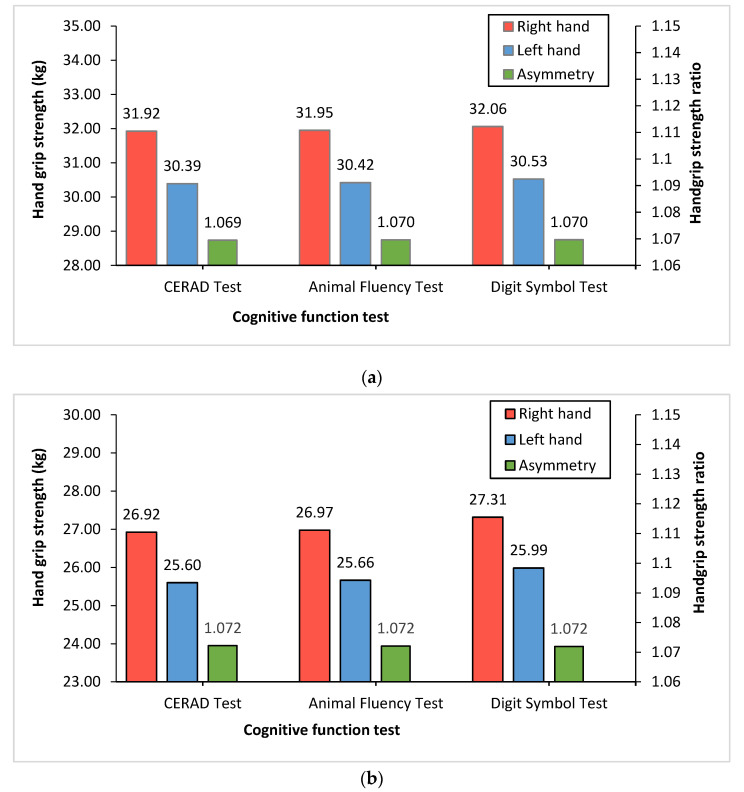
Right and left HGS and handgrip ratio according to cognitive function tests. The red (right hand) and blue (left hand) bars shown in this figure represent the mean of HGS (kg). The green bars (asymmetry) shown in figure represent HGS ratio (dominant HGS (kg)/non-dominant HGS (kg)): (**a**) 60–69 year-old group; (**b**) ≥70 year-old group.

Figure 1 shows each single HGS (kg) and HGS ratio in both hands according to the cognitive function tests. Overall, the right hand by age group had a higher mean grip strength than the left hand in the CERAD test (31.92:26.92 vs. 30.39:25.60), AFT (31.95:26.97 vs. 30.42:25.66), and DSST (32.06:27.31 vs. 30.53:25.99). However, there were no differences in the HGS ratio between cognitive function tests in either age group.

### 3.3. Correlation among HGS and Cognitive Function

Table 2 shows Pearson correlation coefficients among HGS and cognitive function tests. The AFT was significantly correlated with right and left HGS in the 60–69 year-old group (*r* = 0.081) and the ≥70 year-old group (*r* = 0.187). The CERAD test (*r* = 0.080) and the DSST (in right hand *r* = 0.104, and in left hand *r* = 0.101) showed a statistically significant correlation with single HGS test, excluding the relationship between the CERAD test and right HGS in the ≥70 year-old group. Statistically significant positive correlation coefficients were observed between all cognitive function tests and the HGS ratio within the two age groups (each of the tests in 60–69 year-old, *r* = 0.052, 0.064, and 0.070, and in ≥70 year-old *r* = 0.096, 0.073, and 0.068). Compared with the correlation coefficients of the 60–69 year-old group, all correlation coefficients among HGS and cognitive function tests increased in the ≥70 year-old group.

### 3.4. The Estimated Beta Coefficients (SE) for the HGS

Table 3 indicates the estimated beta coefficients (SE) for the HGS in the age groups using cognitive function tests. There was a significant association between AFT and HGS before adjustment. In the DSST, a significant association was only observed in the ≥70 year-old group for the right (beta = 0.247, SE = 0.054; *p* < 0.0001) and left (beta = 0.243, SE = 0.053; *p* < 0.0001) HGS, whereas there was no association between the CERAD test and HGS in the unadjusted model. Compared with the unadjusted model, the fully adjusted beta coefficient for the CERAD test and HGS of both hands were found to be significant in the ≥70 year-old group at 0.022 (SE = 0.008; *p* = 0.0001) and 0.035 (SE = 0.007; *p*-value < 0.0001), respectively. For the AFT, there was no significant association between HGS and the 60–69 year-old group; however, significant beta coefficients were observed in the ≥70 year-old group for both the right (beta = 0.090, SE = 0.034; *p* = 0.0138) and left (beta = 0.108; SE = 0.038, *p* = 0.0074). In the adjusted model, the association between the DSST and HGS in the ≥70 year-old group yielded higher beta coefficients compared to the 60–69 year-old group; most beta coefficients showed an increasing relationship in the older group.

### 3.5. The Estimated Beta Coefficients (SE) for HGS Asymmetry

Table 4 shows the estimated beta coefficients (SE) for HGS asymmetry (dominant/non-dominant handgrip ratio) in the age groups based on cognitive function tests. In the unadjusted model, the beta coefficients for asymmetrical HGS were significantly indicated in the ≥70 year-old group for the CERAD test (beta = −0.337, SE = 0.076) and AFT (beta = −0.841, SE = 0.358) compared to the reference. There was a significant association between DSST and HGS asymmetry in both age groups (60–69 year-olds: beta = −2.620, SE = 0.969; ≥ 70 year-olds: beta = −2.053, SE = 0.990). In contrast, a significant association was observed between the CERAD test and HGS asymmetry in the ≥70 year-old group (beta = −0.226, SE = 0.084) after adjustment.

## 4. Discussion

This present study investigated the association between cognitive function and asymmetrical HGS among older adults in the United States. We found that good cognitive performance was significantly associated with HGS in the ≥70 year-old group. Compared to participants who exhibited symmetric HGS, the cognitive assessment score decreased in the asymmetrical HGS group aged ≥70 years-old. Specifically, the relationship between the CERAD test and asymmetrical HGS remained significant even after controlling for potentially confounding variables. Such information supports our understanding that cognitive function is interrelated with asymmetrical HGS in the elderly population.

Our findings on asymmetrical HGS and cognitive function are partially consistent with the results from previous studies. To the best of our knowledge, only two studies have reported a positive effect of HGS asymmetry on functional decline [26,27]. Collins et al. (2020) provided evidence of a significant association between asymmetrical HGS and weakness due to functional limitations. In a cross-sectional study of 2689 community-based individuals (≥60 years-old), people with handgrip weakness alone (odds ratio (OR): 2.47, 95% CI: 1.14–5.35) and both asymmetrical HGS and weakness (OR: 3.93, 95% CI: 1.18–13.07) had an increased risk of functional limitations, compared to those with symmetrical HGS and were not weak; however, only asymmetrical HGS was not significantly associated with functional limitations (OR: 0.80, 95% CI: 0.62–1.03) [26]. In a panel study by McGrath et al. (2020), 17,163 American adults (≥65 years-old) with asymmetrical HGS and weakness had lower cognitive functioning. Compared to those with symmetrical HGS and no weakness, each group of participants with asymmetrical HGS and weakness exhibited lower cognitive function. The authors suggested that asymmetrical HGS resulted in an approximately two-fold higher risk factor for reduced cognitive functioning in dominant HGS (OR: 1.89, 95% CI: 1.39–3.20) and non-dominant HGS (OR: 2.10, 95% CI: 1.37–3.20) [27].

However, the mechanism underlying the relationship between HGS and cognition remains unclear. One possible explanation is that aging adults with cognitive impairment may be physically frail, with lower grip strength and walking speed [29]. Moreover, lower cognitive function may be related to reduced muscle strength and mass [30,31,32]. Cognitive impairment may result in reduced physical activity [33,34,35], leading to loss of muscle mass. Additionally, several causes of the relationship between a decline in cognitive function and muscle strength and mass have been reported such as inflammation [36,37,38], oxidative stress [39,40,41], and myokines [42]. Another explanation is that neuronal degeneration causes a lack of cognitive capacity [43], and it may also physically decrease muscle strength. For example, neurophysiological changes in age-related geriatric problems result in decreased muscle mass and strength and degenerate inadequate motor performance and neuromuscular junction activity [44]. Moreover, there are pathological characteristics, including biochemical and neuroanatomical alterations, synaptodegeneration, cell loss, neurotrophic failure, cellular genetics, neuronal selective vulnerability, and other factors that develop in the brains of patients with mild cognitive impairment [43]. 

To the best of our knowledge, this study is the first to demonstrate an association between asymmetrical HGS and cognitive function. We analyzed the data from the NHANES study, which is a large-scale and powerful study, and included potential covariates to establish the independence of the relationship between them. However, this study has several limitations. First, as this was a cross-sectional study, these associations could not be examined for causality. Therefore, it is difficult to suggest that the results of this study generalize the causal relationship between asymmetrical HGS and cognitive function. Second, the study was not free from bias since it included self-reported data. Because this study was based on an observational investigation, recall bias remains in the characteristics of participants, so we cannot rule out residual confounding effects from unmeasured confounders. Moreover, some variables affecting cognition, such as dietary intake, medication, and occupation, were not analyzed in the statistical model.

## 5. Conclusions

Our study found that low cognitive function was related to asymmetrical HGS in participants aged 60 years or older in the United States. Contrastingly, symmetrical HGS was associated with good cognitive performance. These findings suggest that the HGS may be a valuable indicator of cognitive impairment. Asymmetrical HGS may be an indirect marker for cognitive decline, independent of confounding factors in the older population. However, further research is required to replicate our results and establish the possible mechanisms for cognitive function and asymmetrical HGS.

## Figures and Tables

**Table 1:** Characteristics of the study population according to age groups.

	60–69 Years-Old (*n* = 1446)		≥70 Years-Old (*n* = 1283)		*p*-Value
**Sex**					0.6618
Male	703	(53.4)	613	(46.6)	
Female	743	(52.6)	670	(47.4)	
**Race/Ethnicity**					<0.0001
White	506	(38.7)	801	(61.3)	
Black	422	(65.4)	223	(34.6)	
Hispanic	362	(70.2)	154	(29.8)	
Others	156	(59.8)	105	(40.2)	
**Income, USD**					0.1979
<20,000	362	(50.9)	349	(49.1)	
≥20,000	1084	(53.7)	934	(46.3)	
**Cigarette smoking**					<0.0001
Current smoker	257	(73.9)	91	(26.2)	
Former smoker	507	(48.8)	532	(51.2)	
Never smoker	682	(50.8)	660	(49.2)	
**Alcohol drinking**					<0.0001
Drinker	1024	(55.7)	813	(44.3)	
Non-drinker	422	(47.3)	470	(52.7)	
**Moderate recreational activities for at least 10 min**					0.0126
Yes	588	(56.0)	462	(44.0)	
No	858	(51.1)	821	(48.9)	
**Body Mass Index (kg/m^2^)**					0.0047
Underweight (<18.5)	19	(46.3)	22	(53.7)	
Normal weight (18.5–24.9)	358	(51.1)	342	(48.9)	
Overweight (25–29.9)	477	(49.9)	478	(50.1)	
Obese (≥30)	592	(57.3)	441	(42.7)	
**Diabetes**					0.9511
Yes	361	(53.1)	319	(46.9)	
No	1085	(53.0)	964	(47.1)	
**Hypertension**					<0.0001
Yes	840	(49.3)	864	(50.7)	
No	606	(59.1)	419	(40.9)	

**Table 2:** Pearson correlation coefficients between HGS and cognitive function tests according to age groups.

Pearson Correlation Coefficients (*p*-Value)						
	60–69 Years-Old			≥70 Years-Old		
	CERAD Test	AFT	DSST	CERAD Test	AFT	DSST
Right hand	−0.033 (0.2006)	0.081 (0.0018)	0.002 (0.9519)	0.047 (0.0846)	0.187 (<0.0001)	0.104 (0.0002)
Left hand	−0.36 (0.1647)	0.079 (0.0022)	−0.015 (0.5637)	0.080 (0.0033)	0.192 (<0.0001)	0.101 (0.0003)
Asymmetric	0.052 (0.0358)	0.064 (0.0093)	0.070 (0.0048)	0.096 (0.0002)	0.073 (0.0051)	0.068 (0.0110)

Abbreviation: CERAD = Consortium to Establish a Registry for Alzheimer’s Disease, AFT = Animal Fluency Test, and DSST = Digit Symbol Test.

**Table 3:** Beta coefficients (SE) for HGS in age groups by cognitive function test.

	Unadjusted				Adjusted ^a^			
	60–69 Years-Old		≥70 Years-Old		60–69 Years-Old		≥70 Years-Old	
	Beta (SE)	*p*-Value	Beta (SE)	*p*-Value	Beta (SE)	*p*-Value	Beta (SE)	*p*-Value
CERAD test								
Right hand	−0.004 (0.004)	0.3475	0.006 (0.006)	0.3319	0.011 (0.008)	0.1849	0.026 (0.006)	0.0001
Left hand	−0.005 (0.005)	0.2630	0.010 (0.006)	0.1074	0.009 (0.008)	0.2489	0.035 (0.007)	<0.0001
AFT								
Right hand	0.046 (0.020)	0.0266	0.118 (0.023)	<0.0001	0.055 (0.029)	0.0628	0.090 (0.034)	0.0138
Left hand	0.040 (0.019)	0.0454	0.124 (0.022)	<0.0001	0.033 (0.034)	0.3512	0.108 (0.038)	0.0074
DSST								
Right hand	0.003 (0.042)	0.9415	0.247 (0.054)	<0.0001	0.308 (0.075)	0.0003	0.243 (0.053)	<0.0001
Left hand	−0.042 (0.038)	0.2793	0.243 (0.053)	<0.0001	0.342 (0.083)	0.0003	0.322 (0.082)	0.0004

^a^ This result was adjusted for age, sex, ethnicity, income, smoking status, alcohol drinking, physical activities, BMI, the presence of diabetes, and hypertension. Abbreviation: SE = standard error, CERAD = Consortium to Establish a Registry for Alzheimer’s Disease, AFT = Animal Fluency Test, and DSST = Digit Symbol Test.

**Table 4:** Beta coefficients (SE) for HGS asymmetry in age groups by cognitive function test.

	Unadjusted				Adjusted ^a^			
	60–69 Years-Old		≥70 Years-Old		60–69 Years-Old		≥70 Years-Old	
	Beta (SE)	*p*-Value	Beta (SE)	*p*-Value	Beta (SE)	*p*-Value	Beta (SE)	*p*-Value
CERAD test								
Symmetric	Reference		Reference		Reference		Reference	
Asymmetric	−0.211 (0.125)	0.1248	−0.337 (0.076)	<0.0001	−0.174 (0.121)	0.1601	−0.226 (0.084)	0.0117
AFT								
Symmetric	Reference		Reference		Reference		Reference	
Asymmetric	−0.636 (0.401)	0.1226	−0.841 (0.358)	0.0251	−0.066 (0.402)	0.8709	−0.384 (0.370)	0.3071
DSST								
Symmetric	Reference		Reference		Reference		Reference	
Asymmetric	−2.620 (0.969)	0.0109	−2.053 (0.990)	0.0462	−1.178 (0.776)	0.1387	−0.491 (1.005)	0.6282

^a^ This result was adjusted for age, sex, ethnicity, income, smoking status, alcohol drinking, physical activities, BMI, the presence of diabetes, and hypertension. Abbreviation: SE = standard error, CERAD = Consortium to Establish a Registry for Alzheimer’s Disease, AFT = Animal Fluency Test, and DSST = Digit Symbol Test.

## Data Availability

The data that support the findings of this study are openly available in (Survey data and documentation) at https://www.cdc.gov/nchs/nhanes/ (accessed on 16 March 2021), reference number [28].
